# Tuberculosis‐associated chylothorax revealing an enlarged lymphatic duct due to tuberculosis lymphadenitis

**DOI:** 10.1002/rcr2.600

**Published:** 2020-06-18

**Authors:** Kojiro Honda, Takeshi Saraya, Chika Miyaoka, Kazuharu Suda, Masachika Fujiwara, Haruyuki Ishii

**Affiliations:** ^1^ Department of Respiratory Medicine Kyorin University School of Medicine Mitaka City Tokyo Japan; ^2^ Department of General surgery Kyorin University School of Medicine Mitaka City Tokyo Japan; ^3^ Department of Pathology Kyorin University School of Medicine Mitaka City Tokyo Japan

**Keywords:** Chylothorax, lymphatic duct, positron emission tomography–computed tomography, tuberculous lymphadenitis

## Abstract

A 77‐year‐old woman presented to our hospital with complaints of persistent cough and low‐grade fever for two months. On radiological analysis, she had moderate right‐sided pleural effusion with right hilar and subcarinal lymphadenopathies. Thoracentesis showed chylothorax of unknown cause. Bronchoscopy revealed a non‐specific inflammatory process. However, thoracoscopic surgery demonstrated a curiously enlarged lymphatic duct with its proximal portion compressed by subcarinal lymphadenopathies, pathologically diagnosed as granulomatous lymphadenitis. Hence, tuberculous lymphadenitis was proven to be the cause of chylothorax. Interestingly, cauterization of the lymphatic duct decreased the total amount of right‐sided pleural effusion along with a change in colour from milky yellow to red. These were in favour of tuberculosis (TB)‐associated chylothorax with the advent of the TB pleuritis. All symptoms and pleural effusion disappeared after the initiation of anti‐tuberculous drugs. The present case showed definite evidence of TB‐associated chylothorax development mechanism via compression of the lymphatic duct by mediastinal lymphadenopathies.

## Graphical abstract

We report a case of chylothorax due to tuberculosis lymphadenitis with distinct evidence of enlarged lymphatic duct macroscopically.

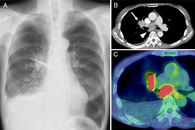

## Introduction

Chylothorax is a rare manifestation of tuberculosis (TB) [1] and lymph node TB is an exceptional aetiology of chylothorax. Herein, we report a case of chylothorax due to TB lymphadenitis with distinct evidence of enlarged lymphatic duct macroscopically.

## Case Report

**Figure 1:**
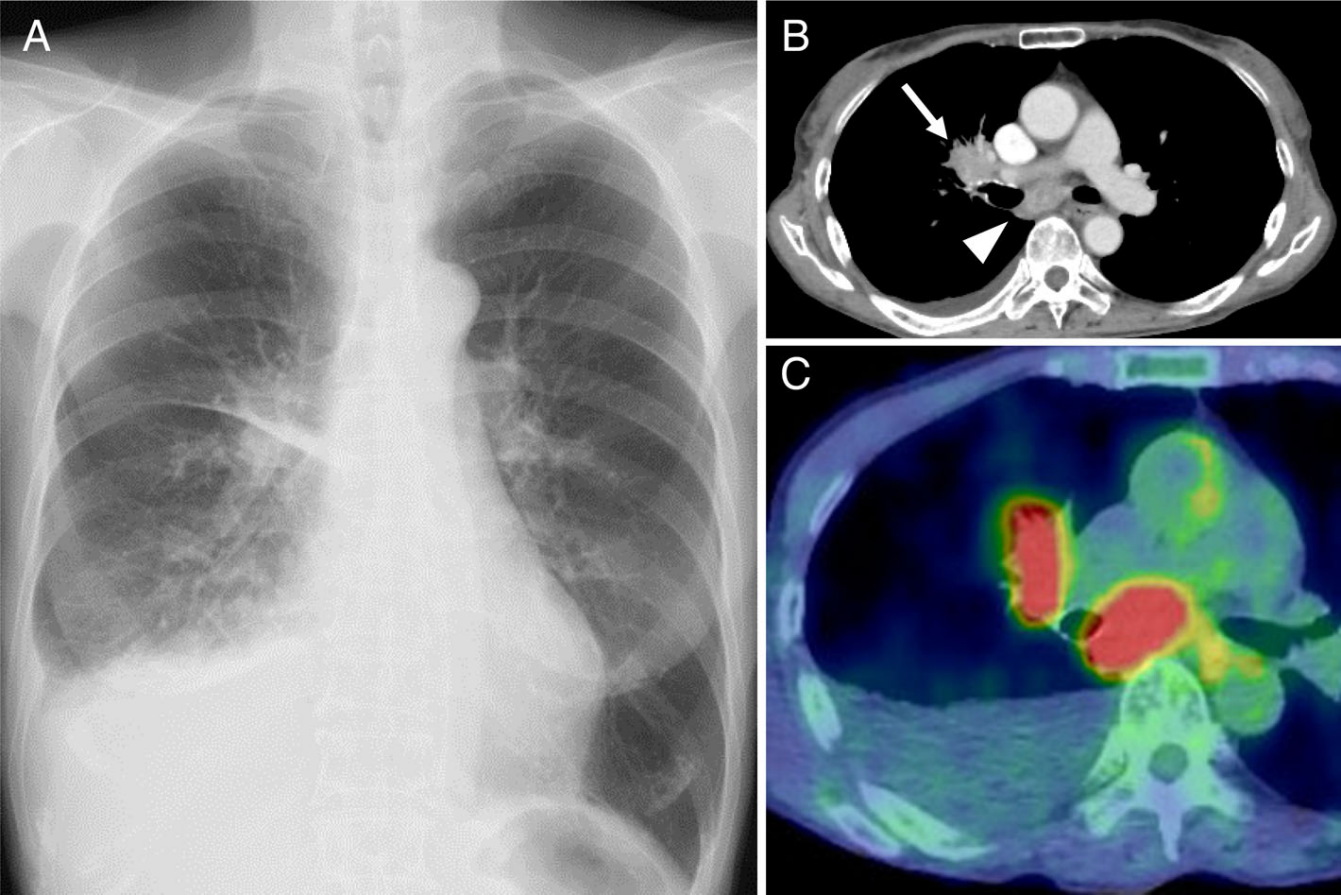
Chest X‐ray taken at the first visit depicted accumulation of moderate pleural effusion at the right side with thickening of the minor fissure together with right hilar lymphadenopathies (A). Thoracic computed tomography (CT) demonstrated a homogeneous enhanced mass measuring 3 cm in diameter (B, arrow) with subcarinal lymphadenopathies (C, arrow head). The fluorodeoxyglucose (FDG)–positron emission tomography (PET)–CT demonstrated a high FDG uptake in right hilar and subcarinal lymphadenopathies (C).

A 77‐year‐old woman presented to our hospital with complaints of persistent cough and low‐grade fever for two months. Her medical history revealed glaucoma and osteoporosis seven years previously. Her vital signs were normal except for mild tachypnoea (respiratory rate, 24 breaths/min) and fever at 38.2°C. Physical examination showed decreased breath sounds at the right middle to lower lung fields. Chest X‐ray taken at the first visit showed moderate right‐sided pleural effusion and right hilar lymphadenopathies (Fig. 1A) confirmed by enhanced thoracic computed tomography (CT), which revealed a homogeneous enhanced mass as large as 3 cm in diameter (Fig. 1B, arrow) with subcarinal lymphadenopathies (Fig. 1B, arrow head). There was no calcification both in right hilar/mediastinal lymphadenopathies and in lung parenchyma.

Serum laboratory data were normal except for mild elevation in white blood cell count at 17,000/μL (cell differential of neutrophil 63.3%, eosinophil 1.1%, basophil 0.8%, monocyte 13.3%, and lymphocyte 21.5%) and C‐reactive protein at 1.19 mg/dL. Other data were normal; total protein (TP) of 7.1 g/dL, lactate dehydrogenase (LDH) of 217 IU/L, total cholesterol (T‐cho) of 202 mg/dL, and triglyceride of 71 mg/dL. An interferon‐gamma release assay was positive.

**Figure 2:**
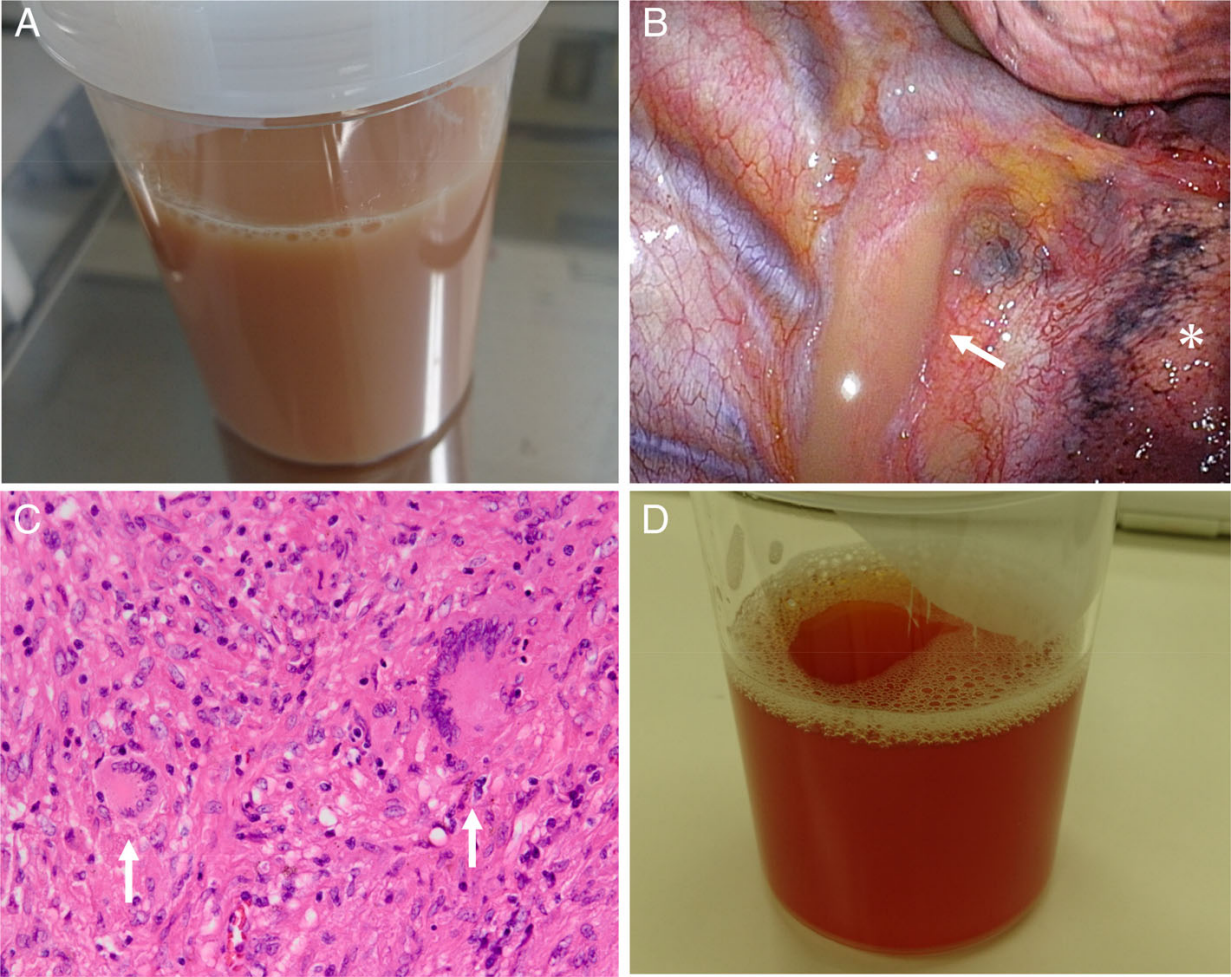
Chylous pleural fluid with a milky yellow appearance (A). A remarkably dilated milky yellow coloured lymphatic duct (arrow) was noted (B), with its proximal portion seemingly compressed by the adjacent enlarged subcarinal lymphadenopathies (B, asterisk). On the haematoxylin–eosin stain, biopsied specimen obtained from the subcarinal lymph node showed epithelioid cell granulomas containing Langhans giant cells (C, arrows). After cauterization of the lymphatic duct, pleural fluid changed in colour to red (D).

Although thoracentesis of the right side was milky yellow in appearance (Fig. 2A), no pathogens were isolated including Mycobacterium, and polymerase chain reaction (PCR) for TB or *Mycobacterium avium*‐complex (MAC) was also negative. No malignant cells were identified (class II). Chemical analysis revealed exudative pleural effusion (LDH 126 IU/L and TP 4.2 g/dL) as well as mild elevation of total cell count (1500/μL), predominantly lymphocytes (89%) followed by neutrophils (3%). Interestingly, the level of triglyceride (TG) in pleural fluid was markedly elevated (713 mg/dL), while the values of adenosine deaminase (ADA) (31.7 IU/L) and glucose (107 mg/dL) were normal. She thus met the criteria of chylothorax of TG greater than 110 mg/dL and a pleural to serum T‐cho ratio less than 1 (0.48).

On the basis of the suspicion of a malignant disease‐induced chylothorax, fluorodeoxyglucose–positron emission tomography–CT (FDG–PET–CT) was performed. The FDG–PET–CT demonstrated high FDG uptake in the right hilum, with a maximum standardized uptake value (SUV) of 7.04, and in the subcarinal lymphadenopathies, with a value of 9.65 (Fig. 1C).

Two weeks after the first visit, bronchoscopy was performed. Endobronchial ultrasound‐guided transbronchial needle aspiration taken from the subcarinal lymph node showed necrotic tissue with abundant neutrophils or monocytes, which was of no diagnostic value. All retrieved respiratory samples were negative for bacterial or acid‐fast culture or PCR methods on TB and MAC.

One month later, her symptoms and moderate right‐sided pleural effusion persisted; thus, thoracoscopic surgery was performed. Curiously, a noticeable enlarged milky yellow lymphatic duct was found (Fig. 2B, arrow) with its proximal portion seemingly compressed by the adjacent enlarged subcarinal lymphadenopathies (asterisk). Biopsied specimens obtained from the subcarinal lymph node showed epithelioid cell granulomas containing Langhans giant cells (Fig. 2C, arrows) along with necrosis, fibrosis, fibrous venous occlusion, and lymphoid hyperplasia. Although no acid‐fast organisms were detected by the Ziehl–Neelsen stain, these findings suggested mycobacterial lymphadenitis associated with TB, she was thus diagnosed with TB lymphadenitis‐associated chylothorax. During the thoracoscopic surgery, cauterization of the lymphatic duct was performed, which resulted in a partial resolution of the pleural effusion. The patient rejected treatment for tuberculous lymphadenitis, and four months later (six months after the first visit), the amount of right pleural effusion had rapidly increased again. Thoracentesis found that the colour of the pleural fluid had changed from milky yellow to red (Fig. 2D) together with an elevation of ADA 77 IU/L and LDH 472 IU/L, suggestive of tuberculous pleuritis. Finally, she was treated with anti‐tuberculous drugs, which led to the complete resolution of symptoms, right‐sided pleural effusion, and mediastinal lymphadenopathies.

## Discussion

The present case had an extremely rare presentation in that an enlarged lymphatic duct compressed by TB lymphadenitis provoked chylothorax in the absence of pulmonary TB, but after cauterization of the lymphatic duct, tuberculous pleuritis emerged as a red effusion.

In general, the aetiology of chylothorax is divided into traumatic and non‐traumatic diseases. Non‐traumatic causes are reported to be 39–72% of all chylothoraces, mainly of malignant origin such as lymphoproliferative disorders (e.g. lymphoma) and haematological and solid tumours [2]. However, the exact incidence of TB‐associated chylothorax is unknown and only 37 cases have been reported so far [1].

Regarding the possible pathogenesis of TB‐associated chylothorax, previous reports have described the following three hypotheses [1]: (1) mediastinal adenopathy compresses the thoracic duct, which leads to leakage of chyle into the pleural space [3, 4]; (2) abdominal lymphadenopathy occludes the cisterna chyli leading to the formation of lymphaticovenous anastomosis [5]; and (3) reduced drainage of lymph due to constrictive pericarditis. The present case supported hypothesis (1), with definite evidence of an enlarged occluded lymphatic duct by tuberculous lymphadenitis in addition to a change in colour of the pleural fluid after cauterization of the lymphatic duct. Rajagopala et al. [1] reported that mediastinal lymph nodes attributed to 51.4% of all TB‐associated chylothorax, as was the present case.

In this case, the timing of the initial onset of the tuberculous pleuritis cannot be determined; however, TB lymphadenitis seemed to gradually progress to the pleura, supported by the findings of elevated pleural ADA and LDH throughout the clinical course.

### Disclosure Statement

Appropriate written informed consent was obtained for publication of this case report and accompanying images.
